# Rapid prototyping of 3D Organic Electrochemical Transistors by composite photocurable resin

**DOI:** 10.1038/s41598-020-70365-8

**Published:** 2020-08-07

**Authors:** Valentina Bertana, Giorgio Scordo, Matteo Parmeggiani, Luciano Scaltrito, Sergio Ferrero, Manuel Gomez Gomez, Matteo Cocuzza, Davide Vurro, Pasquale D’Angelo, Salvatore Iannotta, Candido F. Pirri, Simone L. Marasso

**Affiliations:** 1 Chilab-Materials and Microsystems Laboratory, Department of Applied Science and Technology (DISAT), Politecnico Di Torino, Via Lungo Piazza d’Armi 6, 10034 Chivasso (Turin), Italy; 2 Center for Sustainable Future Technologies, Italian Institute of Technology, Via Livorno, 60, 10144 Turin, Italy; 3 Institute of Materials for Electronics and Magnetism, IMEM-CNR, Parco Area delle Scienze 37/A, 43124 Parma, Italy

**Keywords:** Condensed-matter physics, Materials for devices, Nanoscale materials, Electrical and electronic engineering, Biosensors, Electronic properties and materials, Chemical engineering

## Abstract

Rapid Prototyping (RP) promises to induce a revolutionary impact on how the objects can be produced and used in industrial manufacturing as well as in everyday life. Over the time a standard technique as the 3D Stereolithography (SL) has become a fundamental technology for RP and Additive Manufacturing (AM), since it enables the fabrication of the 3D objects from a cost-effective photocurable resin. Efforts to obtain devices more complex than just a mere aesthetic simulacre, have been spent with uncertain results. The multidisciplinary nature of such manufacturing technique furtherly hinders the route to the fabrication of complex devices. A good knowledge of the bases of material science and engineering is required to deal with SL technological, characterization and testing aspects. In this framework, our study aims to reveal a new approach to obtain RP of complex devices, namely Organic Electro-Chemical Transistors (OECTs), by SL technique exploiting a resin composite based on the conductive poly(3,4-ethylenedioxythiophene):polystyrene sulfonate (PEDOT:PSS) and the photo curable Poly(ethylene glycol) diacrylate (PEGDA). A comprehensive study is presented, starting from the optimization of composite resin and characterization of its electrochemical properties, up to the 3D OECTs printing and testing. Relevant performances in biosensing for dopamine (DA) detection using the 3D OECTs are reported and discussed too.

## Introduction

3D Stereolithography (SL), belonging to the larger 3D Printing or Additive Manufacturing (AM) family, has delivered a new conceptualization of object design and fabrication with beneficial implications also for Organic Electronics^1–3^. When a new technology frontier approaches, researchers are stimulated to push their effort to overcome its limitations. The translation of this concept for SL is the development of new architectures and materials able to add functionalities, and hence implementing and validating new smart objects^4^ and devices^5^ with enhanced performances^6^ and unconventional properties^7^. Three-dimensional expansion of devices brings new solutions and capabilities integration, providing improvements in many fields of application: sensors for wearable electronics^8^, energy storage in building^9^ and biomedical devices for medicine^10^. In the latter case, a class of organic materials showing peculiar properties of biocompatibility and conformability, along with a mixed ionic-electronic charge transport, was developed. Emerging applications are focused on neuromorphic devices^10^, biosensors^11–13^ and cell monitoring^14^. Among these devices, Organic Electro-Chemical Transistors (OECTs) show multifunctional operation in so far as they allow implementing both a transistors-like and a memristive-like response^15,16^.

Recently, a new composite with electrical conducting properties has been proposed by Scordo et al.^17^. This is a SL resin based on poly(3,4-ethylenedioxythiophene):polystyrene sulfonate (PEDOT:PSS), which provides the conducting properties, and Poly(ethylene glycol) diacrylate (PEGDA), acting as is the photo curable matrix in the composite. Of course, the material alone represents only the first milestone for a device development, since its physical properties should be accurately studied and the processing protocols need to be engineered with the aim of obtaining a significant throughput. The passage from the material synthesis to a practical exploitation often requires some decades. In this respect, an exhaustive example is given by carbon nanotubes and/or graphene that, although at the basis of a large set of device prototypes, still lack of an actual killer application to go out from laboratories. Indeed, the Rapid Prototyping (RP) approach grants the extraordinary opportunity to speed up the passage from the materials’ development to their use in well performing devices, ensuring at the same time a highly repeatable fabrication process. This is another aspect that authors had in mind during the design and developing of the presented experimental activity. On this basis, the present work demonstrates, for the first time, that a RP of 3D OECTs, which in this case have been demonstrated to be efficient in biosensing applications, can be effectively obtained by SL. 3D printing methods for the development of electronic devices actually provide 2D structures (e.g. ink-jet^18^ , aerosol-jet^19^) or 3D structures implemented upon multistep processes^20^ and by Fused Deposition Modeling (FDM) and micro-dispensing^21–23^. This work introduces a non-trivial novelty consisting in demonstrating the fabrication of multi-materials 3D objects, which are intrinsically equipped with specific physical properties and can be manufactured in-line by combining 3D SL techniques and engineered resins.

## Results and discussion

### PEGDA:PEDOT resin

A large number of protocols aimed at enhancing PEDOT:PSS conductivity through the combination of thermal curing and acid treatments are reported in literature^24–26^. H_2_SO_4_ 0.5 M treatment is used to induce a significant structural change on PEDOT:PSS layer, because it removes the excess of PSS counter-ion, forming crystallized nanofibrils^25^. A similar approach was applied in this study to obtain the high conductive agglomerates of PEDOT:PSS starting from the commercial PEDOT:PSS solution eluted with sulfuric acid (hereinafter PEDOT:PSS treated by H_2_SO_4_ will be only referred to as PEDOT). Since PEDOT agglomerates size can affect printing accuracy, a fractionation was carried out by dispersing them in a proper solvent. Two different dispersing solvents, ethanol and H_2_SO_4_ 0.5 M solution, were compared aiming to enhance the fractionation of the PEDOT agglomerates. It was found that the solvent affected also the conductivity of the resin. The polymerized PEGDA:PEDOT resin fractionated using ethanol shows a mean conductivity value of 5 × 10^–2^ S/cm compared to 1 × 10^–3^ S/cm of the one fractionated using H_2_SO_4_. Moreover, an optimal dispersion of PEDOT agglomerates was achieved in ethanol because its low density helps to better suspend them in comparison to acid solution. This optimized treatment allows to encapsulate the PEDOT in a PEGDA 575 matrix following the method previously reported^17^.

**Figure 1:**
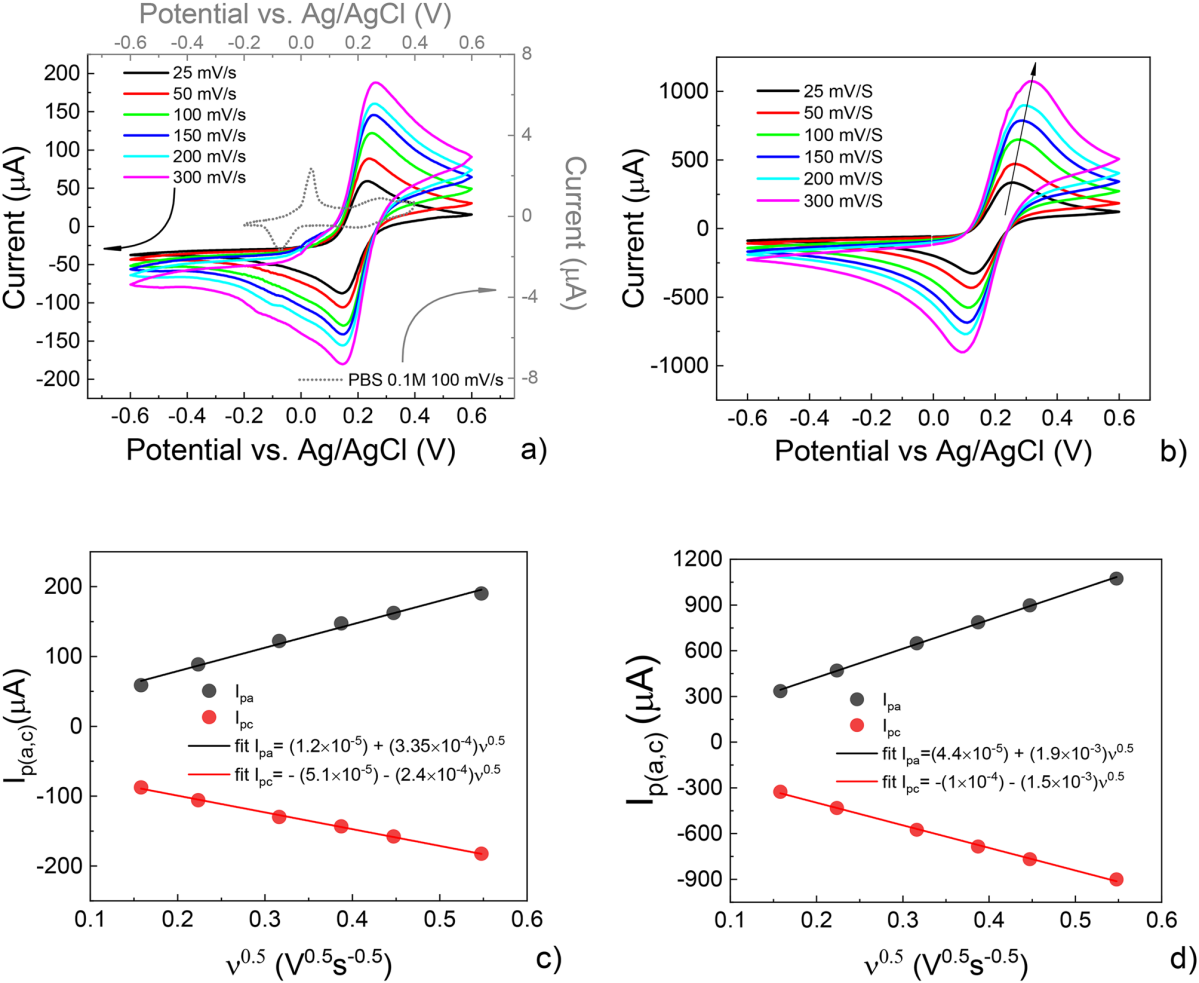
Cyclic voltammetry of potassium ferricyanide (K_3_[Fe(CN)_6_]), 25 mM, in PBS (pH 7.4), 100 mM (scan rates 25, 50, 100, 150, 200 and 300 mV/s) carried out using (**a**) a SPE equipped with Au working and counter electrodes and Ag/AgCl reference electrode; (**b**) the same measurement as in (**a**) using a homologue SPE where the working electrode is covered by a PEGDA:PEDOT layer; (**c**) anodic and cathodic peak positions extracted from (**a**) as a function of the square root of the scan rates (related fitting curves, solid lines); (**d**) anodic and cathodic peak positions extracted from (**b**) as a function of the square root of the scan rates (related fitting curves, solid lines).

The electrochemical behavior of the resin was investigated by Cyclic Voltammetry (CV), carried out using a well-known electrochemically reversible redox system, i.e. the potassium ferricyanide, in PBS (Phosphate-buffered saline) solution. Voltammograms in the case of SPEs (Screen Printed Electrodes) with Working Electrode, (WEs) bare and modified (upon composite resin drop casting) are reported in Fig. 1a, b, respectively. The curve for PBS is also reported, showing a behavior similar to that shown elsewhere^27^.

Figure 1b shows that the electroactive properties of the composite resin are clearly revealed by the amplification of the electrochemical signals acquired in the case of modified WEs. Again, the curve profile is similar to that found elsewhere in the case of potassium ferricyanide in PBS solutions^28,29^.

The enhanced performance for modified SPEs can be rationalized by recurring to the Randles–Sevcik equation:

1 $$ I_{p} = \left( {2.69 \times 10^{5} } \right)AD^{\frac{1}{2}} C\nu^{\frac{1}{2}} , $$

where A is the electrode surface area (cm^2^), D is the diffusion coefficient (cm^2^/s), C is the concentration of the electroactive species in the bulk solution (mol/cm^3^) and ν is the scan rate.

The Randles–Sevick equation describes the effect of ν on the peak current (I_p_) for a reversible process and predicts a linear dependence of the anodic (I_pa_) and cathodic (I_pc_) peak currents with respect to the square root of the scan rate. The linear trends of I_p_ vs. ν^½^ in Fig. 1d indicate a diffusive electrochemical process (i.e. a process controlled by the mass transport velocity of the electroactive species moving towards the WE surface due to a concentration gradient), even if a mild separation of the peak potentials upon increasing the scan rate in the case of modified SPEs (black arrow in Fig. 1b) indicates a slightly lower electron transfer kinetics than that of bare Au WEs. Nevertheless, while a reversible behavior is found for the bare SPE, where the anodic-to-cathodic ratio (I_pc_/I_pa_) is almost 1 (Fig. 1c), the composite resin shows a quasi-reversible behavior. In fact, as predicted by Eq. (1) a linear trend is still obtained, but the composite resin shows an I_pc_/I_pa_ varying from 0.97 (at 25 mV/s) to 0.84 (at 300 mV/s). Values significantly different from 1 are typically associated with chemical reactions coupled with the electron transfer due to the electroactivity of coverages (in our case, this role is ascribable to the PEDOT counterpart).

Taking into account that the surface of the WE is 0.126 cm^2^ and that the K_3_[Fe(CN)_6_] concentration is 2.5 × 10^–5^ mol/cm^3^, a linear fit to the I_pa_ vs. ν½ curve in Fig. 1c allows extracting the diffusion coefficient for potassium ferricyanide in PBS from Eq. (1). In this case D = 1.7 × 10^–6^ cm^2^/s (for instance, the diffusion coefficient of potassium ferricyanide calculated by more sophisticated electrochemical time of flight measurements^30^ is 7.3 ± 0.7 × 10^–6^ cm^2^/s). Using the extracted diffusion coefficient value, an effective surface area of 0.73 cm^2^ is assessed for SPEs covered by PEGDA:PEDOT.

This outcome (i.e. active WE surface upon modification enhanced by a nearly sixfold ratio with respect to that of the bare SPE) demonstrates that the PEGDA:PEDOT resin induces a strongly enhanced electrochemical performance. The hypothesis is that the composite coverage, on one hand enhances the density of redox sites due to the presence of the electroactive component (PEDOT) while, on the other hand, to some extent PEGDA takes the role of PSS in PEDOT:PSS, i.e. it favors the diffusion of ionic species through the PEDOT bulk. This outcome points out the composite resin as ideally suitable for OECTs applications.

### Rapid prototyping of 3D OECTs

The PEGDA:PEDOT resin was optimized to work with a customized SL printer (MICROLA OPTOELECTRONICS s.r.l). The printer works in a top-down configuration and its accuracy is about 80 μm on the XY plane (depending on the resin optical properties) and 50 μm along the vertical axis.

**Figure 2:**
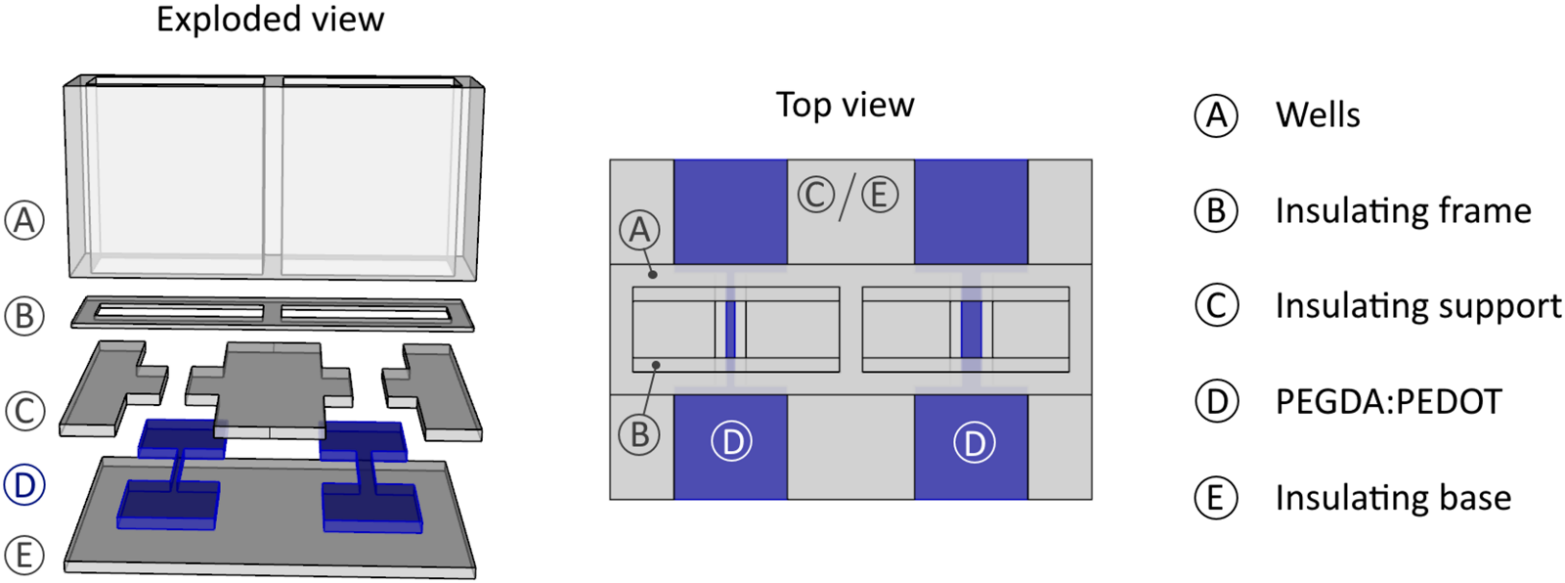
CAD drawing of the OECT to be printed.

The 3D printing of the OECT with the PEDOT-filled resin started from a simple geometry to assess the properties of the resin and its suitability for printing a sensing element. To this end, a 500 μm thick, 2 mm long straight channel was designed. At the channel extremities, two squared contact pads were printed using the same material as the channel^31^. With a view to miniaturized 3D printed sensors, the present OECT was firstly designed to have the smallest channel width achievable with the reported setup and material. Therefore, taking into account the size of PEDOT agglomerates inside PEGDA:PEDOT resin and the printer lateral resolution (around 80 μm), the minimum channel width was set to be 300 μm in order to ensure both accuracy and mechanical stability. A wider channel was also printed (700 μm wide, 2 mm long) to investigate the transistor response at different channel widths. An insulating frame was then added to ensure always the same channel length (2 mm) to be involved in the doping/de-doping process, also ensuring the OECTs response repeatability. Two wells were built, on an additional insulating support, to contain a maximum electrolyte volume of 175 μl. As a result, the 3D printed OECT includes four main parts: an insulating base, the electrically conductive parts, an insulant support for the frame and wells (as reported in Fig. 2). The insulating parts were printed with the commercial SPOTHT resin while the conductive parts with the PEGDA:PEDOT resin. The insulating base, the transistor part and the insulating support were 500 μm thick, while the wells walls were 8 mm high.

Once the device was printed, it was exposed to a thermal curing to post-process the 3D printed part, as previously reported^32^. In addition, this thermal treatment turned out to enhance the 3D OECTs performance with a similar effect observed for the annealing of PEDOT:PSS thin films^33,34^. The thermal treatment was performed by means of a slow heating process followed by a likewise cooling process to gradually release internal stresses and thus avoid undesired fractures.

**Figure 3:**
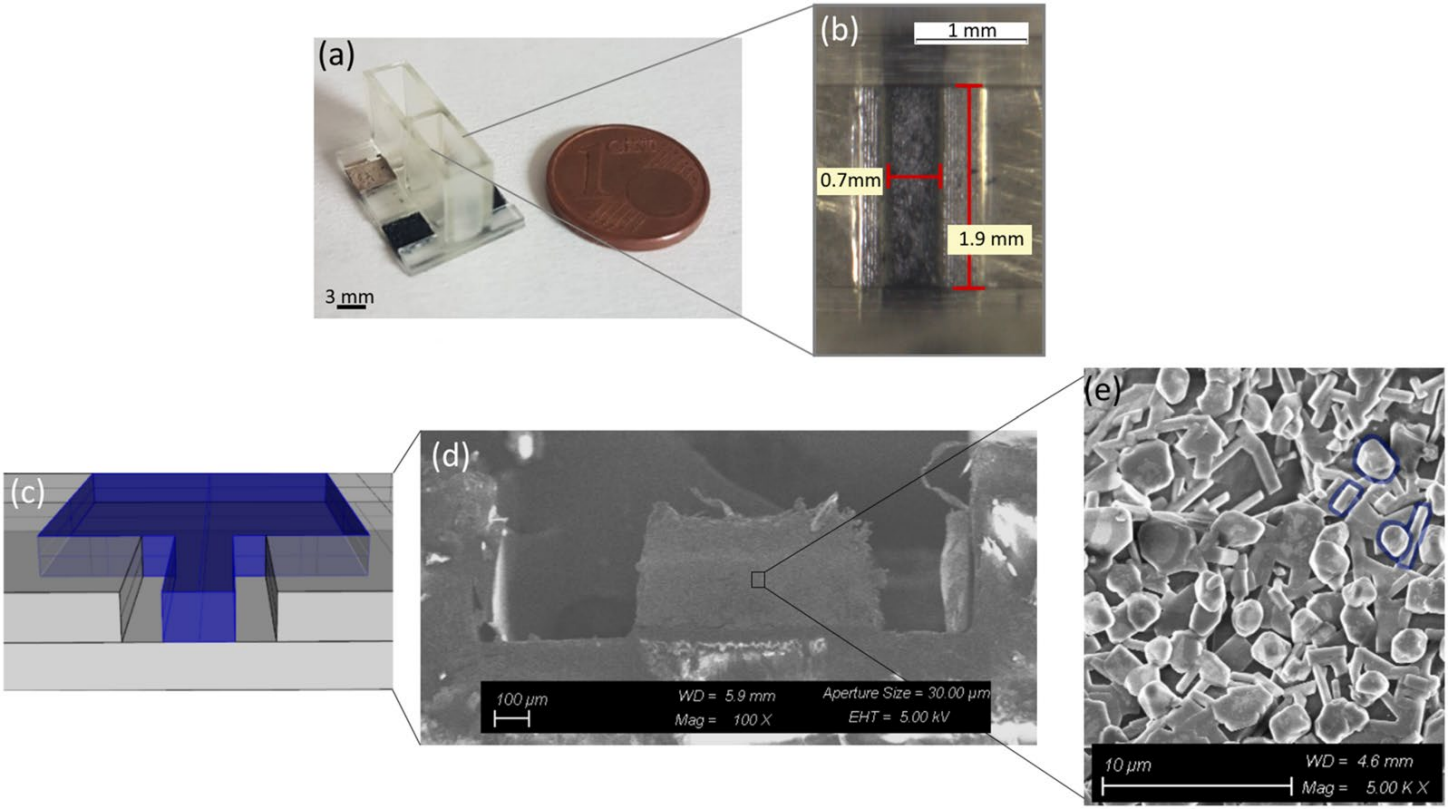
3D printed OECT. (**a**) Device after printing; (**b**) microscope image of the 700 μm wide channel; (**c**) section view of the 700 μm wide channel geometry; (**d**) FESEM image of the same section view reported in (**c**); (**e**) FESEM image of the PEGDA:PEDOT polymerized resin in which the PEDOT particles (some are highlighted in blue) are clearly visible.

The printability of the PEGDA:PEDOT was clearly demonstrated by RP of 3D OECTs with the two different channel widths of 300 μm and 700 μm, respectively (Fig. 3a). The electrical characterization of the final device indicated that the major constrain related to prototyping was not due to the SL intrinsic limitation, but rather to the reproducibility of the OECT response. In fact, the characterization of the 3D printed devices highlighted that the 300 μm wide channel had not a reproducible response like the one by the 700 μm wide channel (Fig. 3b). Therefore, the latter geometry was selected as the best performing one in terms of reproducibility and, hence, considered for the following characterizations. This could be ascribed to the need of a sufficient concentration of active material in the channel in order to properly work as OECT. Nevertheless, the increasing of the PEDOT content in the resin slightly reduces the printability. Hence, to get reproducible and well performing devices the best compromise was achieved using the PEGDA:PEDOT (55:45 wt%) formulation and the 700 μm wide channel.

A section of the OECT channel (as sketched in Fig. 3c) was observed by Field Emission Scanning Electron Microscopy (FESEM) to assess that the insulating SPOTHT resin didn’t accidentally cover the active PEGDA:PEDOT resin, thus passivating it. Indeed, a clear separation between the two materials was observed, as reported in Fig. 3d. Finally, the FESEM imaging also revealed small particles (in the order of a few microns) inside the OECT channel, ascribable to the PEDOT:PSS particles (Fig. 3e).

### 3D OECTs characterization

**Figure 4:**
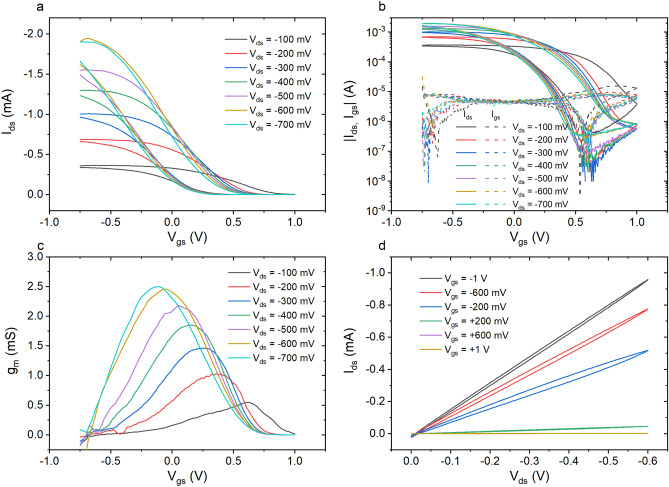
(**a**) Transfer curve measured at different V_ds_; (**b**) logarithmic plot of transfer curve (solid lines) and gate leakage current (dashed lines); (**c**) transconductance curve obtained for V_gs_ scanned from -0.75 V to + 1 V; (**d**) output characteristics of the device.

In order to perform the electrical characterization, the fluidic chamber of the OECT was filled with 150 µL of NaCl 100 mM, while an Ag/AgCl pellet electrode was placed in the electrolyte and exploited as gate electrode. Transfer characteristics (I_ds_ vs V_gs_) were measured at different V_ds_ by sweeping V_gs_ in forward (from − 750 mV to + 1 V) and backward (from + 1 V to − 750 mV) mode (Fig. 4a,b). The drain current decreases upon increasing the gate voltage, as expected for a p-type OECT working in depletion mode^35^. A strong hysteresis is present in the transfer curves due to the diffusion of electrolyte ions inside the PEGDA:PEDOT resin. The I_on_/I_off_ ratio was calculated as the ratio between the current values recorded at V_gs_ = − 750 mV (I_on_) and V_gs_ =  + 1 V (I_off_), respectively, for four different devices. As a result, I_on_/I_off_ was found to increase from $\left( {1.21 \pm 0.40} \right) \times 10^{3}$ at V_ds_ = − 100 mV to $\left( {2.79 \pm 1.6} \right) \times 10^{3}$ at V_ds_ = − 600 mV. The gate current has been continuously monitored throughout the measurements (dashed lines in Fig. 1b) and it was typically two orders of magnitude lower than the drain current in the ON state, with an average value around 10 µA.

Figure 4c shows the differential transconductance $g_{m} = \frac{{\partial I_{ds} }}{{\partial V_{gs} }}$ as a function of V_gs_. The maximum of g_m_ linearly increases from 550 µS at V_ds_ = − 100 mV to 2.5 mS at V_ds_ = − 600 mV where it saturates, while the peak position moves towards negative V_gs_, as expected from the high volume capacitance due to the thick active layer of the device^34,36^. As reported in Table 1, it is expected that maximum transconductance can vary a lot depending on the 3D printing fabrication process, therefore the found values are comparable or even higher with respect to others reported in literature^19,20^, while higher values of g_max_ are achieved for lower thickness^21,37^. In our case, the thickness of the channel is higher than planar one but, the assessed low transconductance value is clearly due to the properties of the resin composite that works on the basis of the PEDOT particles interconnections inside the insulating PEGDA matrix. In addition, high transconductances for OECTs based on PEDOT:PSS have been attributed to the high volumetric capacitance showed by this diphasic organic conductor^38^ . Indeed, the volumetric capacitance in the case of PEDOT:PSS has been attributed to the presence of sulfonate anion-hole pairs, acting as capacitive elements, at the interface between anionic PSS shells and hole-rich PEDOT clusters^39^ . In the case of the resin composite, the PSS phase has been replaced by the PEGDA one and as a consequence capacitive sites with a lower intrinsic capacitance can be attributed to sites constituted by holes (PEDOT) and non-polar groups (PEGDA) at the PEDOT:PEGDA interface.”

**Table 1:** Reported main parameters for 3D printed OECT in literatures: channel dimensions, g_max_, I_ON_/I_OFF_. ND indicates not declared parameter.

Fabrication Techniques	Channel dimension W/L/t (μm)	Materials	gmax (mS)	I_ON_/I_OFF_	References
Stereolithopgraphy	700/1900/500	PEGDA:PEDOT	2.5	2.79 ± 1.6 ⋅10^3^	This work
Aerosol-jet printing	200/200/0.2	PEDOT:PSS	0.52	ND	Ref.^19^
Laser sintering	1,000/15,000/1,000	PEDOT:PSS	2	2	Ref.^20^
FDM/direct writing 3D	1,000/1,600/7.1	PEDOT:PSS	31.8	1.33⋅10^3^	Ref.^21^
Syringe deposition	3,000/3,000/5	PEDOT:PSS	ND	2	Ref.^23^
Melt extrusion/electrospining	200/1,500/200	PEDOT:Nafion	30–40	100	Ref.^38^

The output curves (I_ds_ vs V_ds_) of the device, measured by varying the drain voltage between 0 V and − 600 mV, at a scan rate of 200 mV/s and at fixed gate voltage between − 1 V and + 1 V (Fig. 4d), showed the typical response of a transistor working in linear regime.

The transient behavior of the device has been investigated by holes time of flight (TOF) measurements and by constant gate voltage pulses.

**Figure 5:**
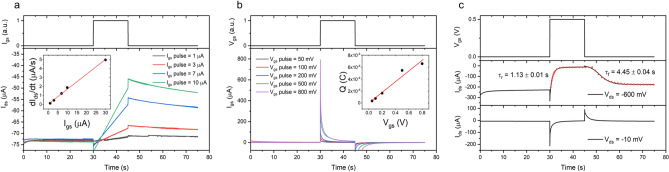
(**a**) Time of flight measurement: device driven by a constant gate current with application of a 15 s pulse with V_ds_ = − 100 mV. The inset shows the linear fit of $$\frac{{dI_{ds} }}{dt}$$ vs I_gs_$$\left( {r^{2} = 0.99} \right)$$ yielding the hole mobility $$\mu = \left( {6.6 \pm 0.2} \right) \cdot 10^{ - 2} \,\,\frac{{{\text{cm}}^{2} }}{{{\text{V}}\,{\text{s}}}}$$. (**b**) Gate current response to a constant gate voltage pulse of 15 s with V_ds_ = 0 V. The charge Q accumulated in the channel has been obtained by integration of I_gs_ curves, the linear fit of Q vs V_gs_$$(r^{2} = 0.98) $$ yielded a capacitance $$C = \left( {890 \pm 60} \right) \mu F$$. (**c**) I_ds_ response to a constant V_gs_ pulse of 15 s at V_ds_ = − 600 mV (central panel) and V_ds_ = − 10 mV (lower panel). The red dotted line in the central panel is an exponential fit of the switching behavior of the device, yielding a rise time $$\tau_{r} = \left( {1.13 \pm 0.01} \right) \,\,{\text{s}}$$ and a fall time $$\tau_{f} = \left( {4.45 \pm 0.04} \right) \,\,{\text{s}}$$.

Figure 5a shows the I_ds_ response while driving the device with constant gate current pulses of 15 s at fixed V_ds_ = − 100 mV. The linear fit of $\frac{{dI_{ds} }}{dt}$ versus I_gs_$\left( {r^{2} = 0.99} \right)$ shown in the inset yielded a hole time of flight $\tau_{h} = \left( {6.03 \pm 0.19} \right) {\text{s}}$ and an effective hole mobility $\mu = \left( {6.6 \pm 0.2} \right) \times 10^{ - 2} \,\,\frac{{{\text{cm}}^{2} }}{{{\text{V}}\,\,{\text{s}}}}$. The slow recovery of I_ds_ after removal of I_gs_ is associated to a slow back-diffusion of ions from the PEGDA:PEDOT resin in the electrolyte.

In order to extract the volumetric capacitance of the active channel material, the gate current has been measured while applying gate voltage pulses at fixed V_ds_ = 0 V (Fig. 5b). The inset of Fig. 5b shows a linear fit $\left( {r^{2} = 0.98} \right)$ of the charge accumulated in the channel versus the applied gate voltage, yielding a volumetric capacitance $C^{*} = \left( {1.28 \pm 0.09} \right)\,\,\frac{{\text{F}}}{{{\text{cm}}^{3} }}$. This value is one order of magnitude lower than the one reported in literature for PEDOT:PSS^40^, as expected from the substitution of the anionic PSS sites with non-polar PEGDA sites.

Figure 5c shows the I_ds_ response to a V_gs_ pulse of 0.5 V at V_ds_ = − 600 mV (central panel) and V_ds_ = − 10 mV (lower panel).

From Bernards model^35^ the transient drain current can be modeled as:

2 $$ I_{ds} \left( t \right) = I_{SS} \left( {V_{gs} } \right) + {\Delta }I_{SS} \left[ {1 - f\frac{{\tau_{h} }}{{\tau_{i} }}} \right]\exp \left( { - \frac{t}{{\tau_{i} }}} \right) $$

where I_SS_ is the steady state drain current, ∆I_SS_ is the difference between initial and final steady state current, $f$ takes in account the spatial non-uniformity in the de-doping process, $\tau_{h}$ is the hole time of flight and $\tau_{i}$ is the ionic RC time constant.

At low drain voltage (lower panel) a spike and recovery behavior is obtained as expected from Bernards model^35^ for slow hole transport. At high drain voltage the response is dominated by a monotonic decay of the drain current, the exponential fit yielding a rise time $\tau_{r} = \left( {1.13 \pm 0.01} \right) {\text{s}}$ and a fall time $\tau_{f} = \left( {4.45 \pm 0.04} \right) {\text{s}}$. The greater fall time is consistent with the slow recovery obtained during the TOF measurements. The presence of a small spike in the drain current upon application/removal of the gate voltage pulse even at high V_ds_ is attributed to the large source/drain spacing of 2 mm, from the ratio of this spike in drain current versus the corresponding spike in gate current a non-uniformity factor $f \simeq 0.26$ has been calculated^41^.

### Dopamine biosensing

We made an analysis dedicated to the check of performances by the composite resin used in OECTs architectures. This analysis is aimed at giving a tangible example of 3D OECTs suitability in biosensing applications. We hence explored the 3D OECTs efficiency in sensing mode of operation detecting biomolecules of interest in medicine. In the specific case, we implemented the detection, by the 3D OECT, of the dopamine (DA), which is a neurotransmitter regulating the correct functioning of several organs whose loss in some areas of the brain causes, for instance, the Parkinson's disease. The choice of dopamine is also motivated by the fact that, starting from the work by Tang et al.^42^, it is a prototyping molecule in OECTs biosensing^43–46^.

**Figure 6:**
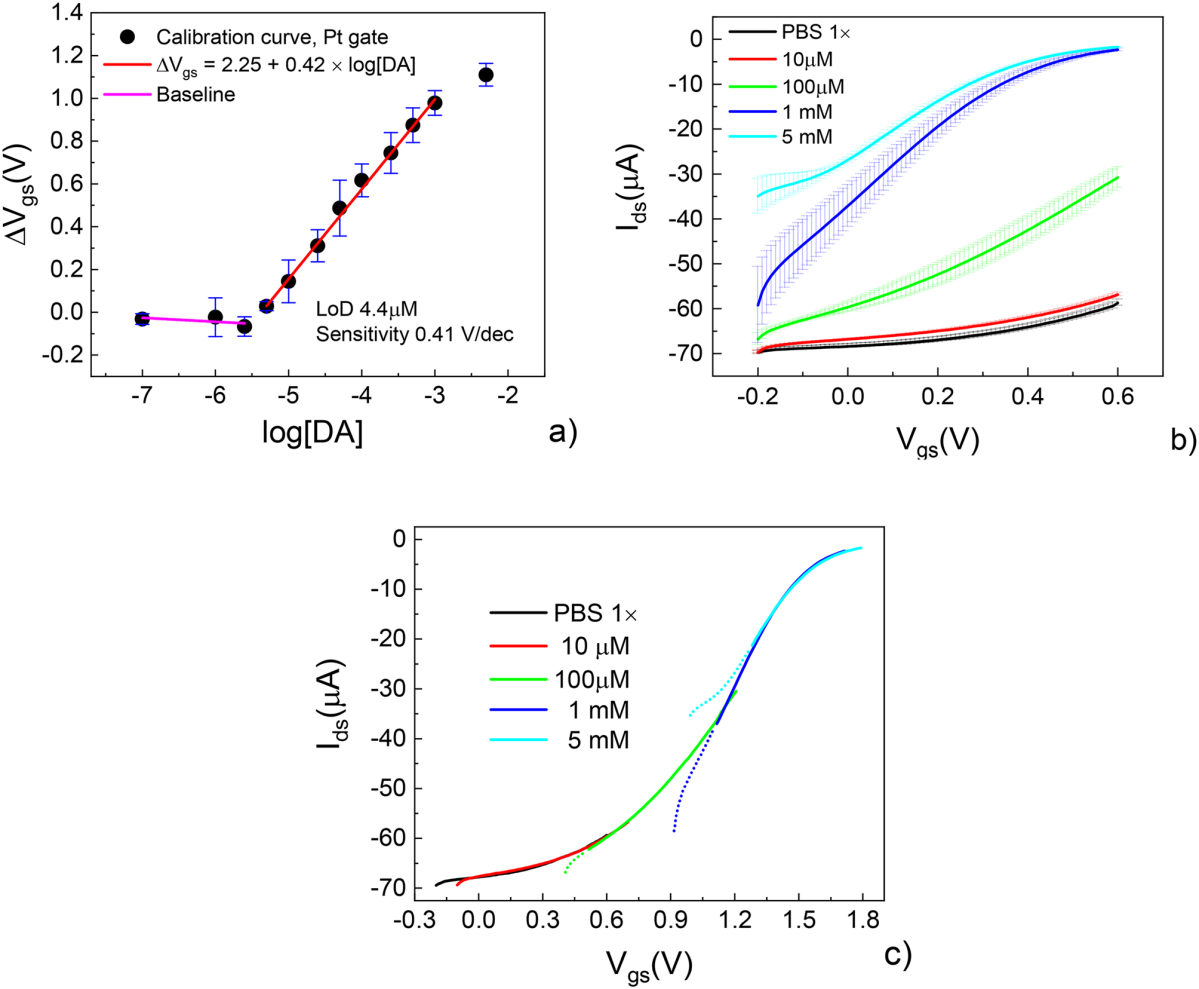
Calibration curve for dopamine sensing by (**a**) a PEGDA/PEDOT 3D OECT; (**b**) typical transfer curves recorded at different dopamine concentrations ([DA] = 0, 10 µM, 100 µM, 1 mM and 5 mM, error bars are standard deviations) and (**c**) universal curve obtained by merging transfer curves in (**c**) upon shifting them along the V_gs_ axis.

The calibration curve for DA detection implemented using a PEGDA:PEDOT OECT and calculated from transfer curves is reported in Fig. 6a. Some selected (averaged) transfer curves at lower and higher DA concentrations ([DA] = 0, 10 µM, 100 µM, 1 mM and 5 mM in PBS 100 mM) and the related universal curve are reported as an example in Fig. 6b,c, respectively. Quasi-reversibility of electrochemical processes in the composite resin is indicated by the large error bars upon averaging repeated measurements, especially at lower V_gs_ (the baseline current, I_ds_ for V_gs_ → 0, shifts towards lower values and the largest shift is systematically recorded after the first measurement).

The basic idea is to exploit the reactivity of catecholamines with a bare Pt electrode, whereas dopamine is expected to be involved in a 2-electrons exchange mechanism with the platinum electrode^47^. The measurement protocol is defined by the scheme used by Tang et al.^42^. The proposed scheme relies on the fact that the reaction of dopamine at the platinum gate electrode is able to modulate the effective gate voltage (V_gs,eff_) due to an offset voltage (V_offset_) strictly connected to the analyte concentration and expressed as^48^:

3 $$ V_{offset} { } = V_{{gs,{ }eff}} - V_{gs} = { }\Delta V_{gs} = { }2.30{ }\left( {1 + \gamma } \right)\frac{{K_{B} T}}{nq}{ }\log \left[ {DA} \right] + cost $$

where K_B_ is the Boltzmann’s constant, T is temperature, n is the number of electrons transferred at the gate, [DA] is the dopamine concentration and γ is the ratio between the capacitances at the gate electrode/electrolyte and electrolyte/active channel interfaces. This equation predicts that an increase of dopamine concentration causes the enhancement of V_offset_ that, in turn, implies a reduction of the channel current.

The device performance can be rationalized by extracting two parameters from the calibration curve, i.e. the Limit of Detection (LoD) and the sensitivity, the former representing the lowest concentration of the analyte that can be reliably distinguished if compared to a blank measurement. Sensitivity, indeed, describes the minimum input parameter able to create a detectable output response by the sensor. The assessment of ΔV_gs_ by evaluating the shift of transfer curves is expected to be less effective in determining lower LoDs if compared to its evaluation based on real-time measurements, such as the recording of the step-like device response upon subsequent injections of the analyte to be detected in an electrolytic reservoir^49,50^. Nevertheless, both devices show a LoD falling in the micromolar range and a dynamic window varying between the micromolar and the millimolar range of [DA]. Assessed LoD is two orders of magnitude higher than that experimentally achievable by analytical tools, such as Cyclic Voltammetry in the case of SPEs having a Pt working electrodes (50 nm^42^), but is still in line with LoD values obtained using OECTs with unmodified Pt gate electrodes^42,45^. However, the OECT operation consists of an electronic transduction (by the active channel) of ionic signals associated to some reactions at the gate electrode/electrolyte interface (as in the case of DA and Pt electrodes) and/or capacitive effects (Electrical Double layer formation). In the former case, the efficiency of such transduction often requires a specific functionalization aimed at amplifying the charge transfer mechanisms at the metallic gate/liquid electrolyte interface, hence LoDs comparable to the lowest DA concentrations determined by CV measurements can only be achieved upon functionalizing the Pt gate electrode^43,51^.

On the contrary, calibration curves show a larger sensitivity if compared to that assessed using different experimental approaches^50^. Specifically, PEGDA:PEDOT OECTs show a markedly enhanced sensitivity of 0.41 V/dec. This enhanced sensitivity may be ascribed to the PEGDA:PEDOT microstructure. In fact, the composite resin microstructure (Fig. 3e) shows a homogeneous dispersion of the PEDOT phase, whose exposure to the electrolyte is not mediated by the PSS phase. This is difficult to achieve with standard PEDOT:PSS, where both solvent^52^ and thermal^34^ annealing cause the PEDOT clusters to be segregated in the bottom part of PEDOT:PSS films and the PSS phase interfaced to the gate electrolyte.

## Conclusion

We demonstrated that even devices as complex as OECTs can be well fabricated by standard SL technique. RP of 3D OECTs was achieved by optimizing a new composite material: the PEGDA:PEDOT resin. The latter is a photocurable composite that we demonstrate to maintain performance comparable to that of PEDOT:PSS in terms of electrical conductance, bulk ionic diffusion mechanism, gating response in OECTs architectures and, hence, showing quite good sensing response. The optimization of this characteristics depends not only on the PEDOT content inside the resin, but also on the active material pre-treatment and the printability, which ultimately affects the OECTs behavior. Finally, 3D OECTs showed an enhanced sensitivity of 0.41 V/dec towards dopamine detection, thus demonstrating a precise assessment of concentrations when used as biosensors and, hence, in a perspective view, the freeform fabrication enabled by 3D printing paves the way for their integration in IoT and smart objects.

## Methods

### PEGDA:PEDOT resin

The highly conductive PEDOT is prepared starting from the commercial solution CLEVIOS PH1000 (HERAEUS) eluted with 0.5 M H_2_SO_4_ at 1:10 ratio (CLEVIOS:0.5 M H_2_SO_4_) for 12 h. Then the PEDOT agglomerates were separated from the initial solution by centrifugation at at 4,000 rpm for 15 min with OHAUS FRONTIERS Centrifuge. The agglomerates were dispersed in ethanol and a 20 min fractionation was performed with IKA ULTRATURRAX. A further separation by centrifugation at 4,000 rpm for 15 min allowed to obtain the final PEDOT agglomerates, which were added to PEGDA-575:IRGACURE 819 (100:1 wt%) resin (MERK) in a ratio PEGDA:PEDOT (55:45 wt%).

### CV measurements

Voltammograms (potential range − 0.6 to 0.6 vs. Ag/AgCl) were acquired using commercial SPEs (C220BT DROPSENS, METROOHM: Au Working Electrode, WE, surface area 0.126 cm^2^, Au Counter Electrode, CE, Ag (AgCl) Reference Electrode, RE) with and without modification of the WE by the resin composite (drop casted, 3 µL), using a PALMSENS4 potentiostat/impedance analyzer. Curves were acquired at different scan rates (ν = 25, 50, 10, 150, 200 and 300 mV/s) in the case of a potassium ferricyanide salt (K_3_[Fe(CN)_6_]) in PBS solution (25 mM in PBS solution, 100 mM, pH 7.4) as electrolyte.

### 3D printing of the device

Two resins were used to print the device: SPOTHT resin from SPOTA materials and PEGDA:PEDOT resin prepared as reported in the previous section. The customized SL printer was set to build up the object by printing a 100 μm thick layer each time and alternating the materials. In the top-down printing configuration, the printer platform moves inside a vat filled with liquid resin, then it comes out and a recoater blade slides on it (at a distance almost equal to the layer thickness). In such step, the resin is made more homogeneous at the polymerization site and is ready to be crosslinked by the exposure through of a UV laser (405 nm wavelength in this case) moved by a galvanometric head. For the 3D printed OECTs, the printing process was slightly modified to allow for the use of different materials. First of all, the insulating base was printed with SPOTHT resin setting the following parameters: 10 mW laser nominal output power, 1,000 mm s^−1^ laser hatch speed, 50 μm hatch spacing. Then, the printing was paused and the printer vat filled with PEGDA:PEDOT resin. The printing was resumed to print the OECT channels and pads, setting the following parameters: 20 mW laser nominal output power, 1,000 mm s^−1^ laser hatch speed, 50 μm hatch spacing. Finally, the insulating support, the insulating frame and the wells were printed after the PEGDA:PEDOT resin was replaced with the SpotHT resin. The printed devices were carefully rinsed with isopropyl alcohol and dried. A final post-curing step was performed in a thermal chamber (BINDER VDL): starting from room temperature, the devices were heated in vacuum up to 120 °C for one hour. Then, they were left cooling in vacuum, until the room temperature was reached again. A ZEISS SUPRA 40 Field Emission Scanning Electron Microscopy (FESEM), electron high tension of 5 kV, was used to investigate the PEDOT agglomerates in the 3D OECTs.

### OECT characterization

Electrical characterization has been performed using a KEYSIGHT B2912A Source Meter precision unit connected to the micromanipulators of a probe station through triax cables and controlled by the proprietary KEYSIGHT software. A solution of 100 mM NaCl in DI water (pH = 7) was used as gate electrolyte during output and transfer-characteristic measurements and during transient response measurements. An Ag/AgCl electrode (1 mm of diameter) was used as gate electrode. Transfer characteristics, i.e., the channel current (I_ds_) as a function of the applied gate voltage applied (V_gs_) at fixed drain-source voltage (V_ds_), were measured at fixed V_ds_ = − 100 mV, − 200 mV, − 300 mV, − 400 mV, − 500 mV, − 600 mV and − 700 mV, by sweeping V_gs_ from − 750 mV to + 1 V and back with a scan rate of 5 mV/s. Output characteristics were measured by sweeping the V_ds_ from 0 V to − 600 mV with a scan rate of 200 mV/s at different fixed V_gs_ of − 1 V, − 600 mV, − 200 mV, + 200 mV, + 600 mV and + 1 V.

Time of flight measurements have been performed driving the device with a 15 s constant gate current pulse of 1 μA, 3 μA, 7 μA, 10 μA and 30 μA at fixed V_ds_ = − 100 mV.

In order to extract the volumetric capacitance, the gate current has been measured at fixed V_ds_ = 0 V while V_gs_ pulses of 15 s were applied at 50 mV, 100 mV, 200 mV, 500 mV and 800 mV.

The response behavior of I_ds_ to a fixed V_gs_ pulse has been investigated by applying a gate voltage pulse of + 500 mV for 15 s while fixing V_ds_ = − 600 mV and V_ds_ = − 10 mV. All the transient response measurements have been performed with a sampling rate of 33.33 Hz.

### Dopamine biosensing

The PEGDA-PEDOT OECT response was evaluated by acquiring transfer characteristics in presence of different concentrations of dopamine (MW = 189.54 mg/mol, Alfa Aeser) in 100 mM PBS solution. All the electrical measurements were performed by varying the gate voltage from − 0.2 to 1 V at fixed V_ds_ = − 0.6 V, with a scan rate of 10 mV/s. The PDMS well was filled with 150 μL of PBS/DA solutions with dopamine diluted at concentrations ranging from 100 nM to 5 mM. A vertical Pt wire gate (0.6 mm diameter, FRANCO CORRADI) was used as the gate electrode. Each measurement was repeated three times and the channel was washed with H_2_O Milli-Q and PBS solution after each measurement step. ΔV_gs_ was calculated by merging transfer curves at different concentrations (three runs per device for each DA concentration, repeated using three different devices) upon shifting them along the V_gs_ axis in order to define a universal curve^49^. Error bars have been calculated as the statistical error of the mean (σ_dev_/N − 1, where σ_dev_ are the standard deviations and N is the number of measured devices).

The device LoD against DA sensing was assessed by estimating the intersection between the slope of the linear fit in the dynamic window (sensor transfer curve) and the ideal baseline for concentrations falling below such window. Sensitivity was calculated as the slope of the sensor transfer curve.
